# Mixed Messages and COVID-19 Prevention: Why Information Is Not Always Enough to Protect Meat Processing Workers

**DOI:** 10.1016/j.focus.2023.100128

**Published:** 2023-06-21

**Authors:** Jacqueline M. Sivén, Julia F. Coburn, Tristan P. Call, Dillon Mahoney, Rebeca Rodríguez Flores, Harpriya Kaur, Michael A. Flynn, Cammie K. Chaumont Menéndez

**Affiliations:** 1 Division of Science Integration, National Institute for Occupational Safety and Health, Centers for Disease Control and Prevention, Cincinnati, Ohio; 2 Centro de los Derechos del Migrante, Inc., Mexico City, Mexico; 3 Bedford County Listening Project, Shelbyville, Tennessee; 4 Department of Anthropology, College of Arts and Sciences, University of South Florida, Tampa, Florida; 5 Centro de los Derechos del Migrante, Inc., Oaxaca, Mexico; 6 Division of Safety Research, National Institute for Occupational Safety and Health, Centers for Disease Control and Prevention, Morgantown, West Virginia

**Keywords:** Vaccine confidence, occupational health, health equity, immigrant workers

## Abstract

**Introduction:**

The objective of this project was to investigate U.S. meat and poultry processing workers’ knowledge of COVID-19; their perceived ability to protect themselves from infection; and perspectives on COVID-19 vaccines to inform COVID-19 prevention efforts within this linguistically, racially, and ethnically diverse workforce.

**Methods:**

Qualitative semistructured in-depth interviews were conducted with Mexican, Central American, Congolese refugee, and Black or African American meat/poultry processing workers from Mississippi, Minnesota, Virginia, and Kentucky (N=40). Data were collected from December 5, 2020 to January 28, 2021. Interview audio was transcribed, and rapid qualitative data analysis was used to analyze transcripts.

**Results:**

Most participants expressed receiving mixed messages about COVID-19 protection measures: they were told how to protect themselves (*n*=38), but workplace policies (such as lack of paid sick leave) often undermined their efforts. Participants who were asked about COVID-19 vaccines (*n*=31) were aware that there were 1 or more vaccines available to protect them from COVID-19; one third were eager to get vaccinated.

**Conclusions:**

Community-based efforts may consider supplementing large-scale unified information campaigns to prevent mixed messages, address worker needs to accurately gauge the threat of illness to their communities, and empower them to prevent infection.

## Highlights

• Meat processing workers reveal difficulties with COVID-19 prevention and reduction measures.
• Workers were concerned about vaccine safety during early vaccine roll out.
• Collaboration with community organizations is the key to understanding community perspectives.
• Workplaces can facilitate or hinder public health efforts in diverse communities.

## INTRODUCTION

At the onset of the pandemic, meat and poultry processing facilities were one of the most visible examples of coronavirus disease 2019 (COVID-19) disease transmission in the workplace,^1^^,^^2^ disparities in COVID-19 exposure and outcomes,^3^ pre-existing disparities in determinants of health,^4^, ^5^, ^6^ and potential threats to the food supply chain.^7^ Public health professionals encountered limited data that would allow them to tailor COVID-19 infection prevention and control strategies for this racially, ethnically, and linguistically diverse workforce.^1^^,^^2^

Frontline workers in meat/poultry processing facilities come from diverse communities primarily represented by linguistic, racial, and ethnic minority groups; migrant communities; and refugee populations.^4^ Operations in meat/poultry processing facilities provide a set of conditions in one work site that coalesces toward optimal COVID-19 transmission: (1) high-contact, congregant settings with a limited ability to physically distance; (2) poorly ventilated, refrigerated environments that may facilitate COVID-19 transmission;^8^ (3) work intensification;^8^ and (4) noisy workspaces that require shouting to communicate.^1^^,^^9^ This workforce experiences a further disproportionate burden of broad social and economic disadvantages.^4^^,^^10^, ^11^, ^12^, ^13^, ^14^, ^15^, ^16^ The barriers to the knowledge of COVID-19 disease transmission and effective protective measures,^11^, ^12^, ^13^, ^14^, ^15^ factors affecting vaccine confidence and hampering vaccine use,^17^, ^18^, ^19^ vary across the different communities that source the frontline workers of this industry.

Our objective was to explore U.S. meat/poultry processing workers’ knowledge of COVID-19, their perceived ability to protect themselves from infection, and their perspectives on COVID-19 vaccines to inform the development of culturally tailored COVID-19 prevention strategies. We provide findings on interviews we conducted across several states with representatives from various communities in this linguistically, racially, and ethnically diverse workforce.

## METHODS

### Study Population

A multidisciplinary team, including academicians, community outreach workers, and public health researchers, conducted qualitative semistructured in-depth interviews with 40 meat/poultry processing workers in Mississippi, Minnesota, Virginia, and Kentucky. The multidisciplinary team was also racially/ethnically diverse (Black, White, Hispanic/Latino, and South Asian), was multinational (U.S. born, foreign born, people with family members who are immigrants), and included those from working-class backgrounds. The diversity of the team benefited the critical discussion of potential assumptions during the analysis process.

Participants were Black or African American (*n*=10), Congolese refugee (*n*=10), Mexican (*n*=10), and Central American (*n*=10; 9 from Guatemala and 1 from Honduras) meat and poultry processing workers (Table 1). Participants were initially recruited through trusted relationships developed by team members before the pandemic and subsequently expanded using snowball sampling. In most cases, team members’ connections to participant communities were strengthened by pre-existing relationships with community-based organizations, including Centro de Los Derechos del Migrante, Inc., whose staff also participated in data collection and analysis. Social media outreach was also conducted to establish contact with some participants. The team's previous research and outreach experiences with Mexican migrants, Central American migrants, and Congolese refugees informed them that these were critical populations to include in the project. Interviews were conducted in English, Spanish, Mixteco, Swahili, Kinyarwanda, and French through phone and web-based platforms. Participants also spoke the following as mother tongues: Mam, Náhuatl, Totonac, Zapotec, and Q'anjob'al. Interviews were conducted by team members in the preferred language(s) spoken by participants without additional interpretation; these were audio recorded and subsequently transcribed and translated into English by the team. Data were collected on December 5, 2020 through January 28, 2021 and captured a snapshot of participants’ knowledge and perspectives during the early days of the COVID-19 vaccine roll out. This study received a nonresearch determination from the IRB of the National Institute for Occupational Safety and Health.

**Table 1:** Demographic and Behavioral Characteristics of Meat and Poultry Processing Workers (N=40)

Characteristic	*n*	%
Age, years		
<25	8	20
25–34	14	35
35–44	15	38
≥45	3	8
Gender		
Male	20	50
Female	20	50
Employment status		
Full time	40	100
Years in the U.S.		
<5 years	7	18
5–9 years	10	25
≥10 years	23	58
Population		
Congolese^a^	10	25
Central American^b^	10	25
Mexican^c^	10	25
Black or African American	10	25
Length of work		
<1 year	8	20
1–5 years	22	55
6–10 years	3	8
>10 years	7	18
Perceived company size		
Large	27	68
Medium	6	15
Small	7	18
How well do you speak English?		
Very well	13	33
Well	3	8
Not well	18	45
Not at all	6	15

a Congolese ethnic groups included 3 Banyamulenge, 2 Banyabuisha, 2 Fulero, 1 Kasongo, and 1 Nyanja; 1 participant did not provide their tribe/ethnicity.

b All Central American participants identified as being of indigenous origin: 8 were Mam from Guatemala, 1 was Q'anjob'al from Guatemala, and 1 was Miskito from Honduras.

c Most Mexican participants identified as being of indigenous origin: 6 were Mixtec, 1 was Nahua, 1 was Totonac, 1 was Zapotec, and 1 was nonindigenous.

### Instrument

Semistructured interviews were conducted after obtaining verbal consent to collect participant narratives concerning the knowledge of COVID-19 and related barriers to safety.^20^ Specifically, the interview guide began by asking about employment location, position, status and tenure, and demographics (age, gender identification, nativity, language spoken, educational attainment, household size, race, and ethnicity). Then, the interviewer asked the participant to describe a typical day at work at the time of the interview and proceeded to ask a set of questions about how the nature of this work has changed since the COVID-19 pandemic began. Specifically, participants were asked how health and safety concerns have changed, how knowledge about COVID-19 has changed, how they protect themselves from being infected with COVID-19, and what is the best way to communicate knowledge to the meat processing workers in their community to protect them from getting the virus. A set of questions was asked about how their employer responded to COVID-19 with respect to workplace safety and administrative measures, the number of shutdowns related to COVID-19 that occurred, and what they wish that their employer had done differently. Toward the end of the interview, the participants were asked how the pandemic and their employers’ responses to it at work now affected their life, what was their life like before the COVID-19 pandemic, how the COVID-19 pandemic affected their community, and whether the participants were ever treated badly at work because of their identity. The participants were asked whether they knew about the vaccine and what their thoughts were about taking the vaccine if it were offered free of charge. Finally, the interview guide ended with asking participants about their well-being: how they are feeling, who they talk to when they are not feeling well, how their workplace affects their well-being, and questions from a standardized questionnaire designed to assess well-being.

### Analysis

A rapid qualitative data analysis methodology was used to analyze transcripts by streamlining and systematizing traditional qualitative methods.^21^ Procedural and interpretative rigor was maintained during analysis through the use of transcripts, condensed transcript summaries, and standardized matrices to streamline thematic coding across participants. Condensed transcript summaries were organized around the domains that were used to create the interview guide and included an “other” category to capture unexpected domains and themes. The domains framing the interview guide were knowledge of COVID-19, population-specific issues/cross-cultural factors, barriers to safety, well-being, power relationships, and vaccines. Data analysis was an iterative process, with themes being reviewed and refined by the data analysis team until a consensus was reached on common themes across the project population. The data collection and analysis team all had previous experience and background knowledge of 1 or more of the project populations and spoke the native language of at least 1 of the project populations (e.g., 1 spoke fluent Swahili, 2 spoke fluent Spanish, all spoke fluent English). In addition, to bring greater coherence to the results and discussion, the authors have used the following terms to identify themes’ prevalence within participant interviews: “most” signifies that theme(s) appear in 80% of interviews or more, “many” signifies that theme(s) appear in between 60% and 79% of interviews, “about half” signifies that theme(s) appear in between 40% and 59% of interviews, and “some” signifies that theme(s) appear in <40% of interviews.

## RESULTS

### Knowledge of COVID-19

Most participants possessed working (albeit incomplete) knowledge about COVID-19, including dangers and preventive measures. Participants described their understanding of how COVID-19 spreads and protective measures at home and work. When asked how they protect themselves from COVID-19, participants most frequently cited cleaning/disinfecting and social distancing; some participants also mentioned staying at home, wearing a mask, and using herbal medicine. Others reported doing nothing. Participants indicated that the best ways to reach meat and poultry processing workers with information about COVID-19 included the use of social media, through in-person outreach, through TV, through their employers, and through the church.

### Seeing Is Believing

Witnessing COVID-19 infection or being personally infected by COVID-19 were pivotal experiences that cemented many participants’ belief that COVID-19 was indeed a serious illness. Although these participants expressed that they did not take COVID-19’s risks seriously at first, their perspective changed after experiencing the virus’ impacts up close. One participant stated, “I knew a lot of people who [were] still partying… but later on, they whole attitude done changed, because when it went killing people close to home, or people at home, your loved ones, they see how serious it is. But at first like, yo, they was taking it as a joke.”

Another participant suggested that lockdown measures were integral in driving home the gravity of the pandemic for him, providing an example of how the ban on sporting events helped to normalize the idea that the pandemic was “real” enough to disrupt major social activities.

### Inability to Protect Themselves From Infection

Most participants observed that the precautionary measures that employers, government agencies, and other authorities prescribed to them to prevent COVID-19 infection routinely went unfulfilled and underenforced, or were impossible to implement. Participants and their coworkers struggled to physically distance, receive hazard pay, and stay at home when sick for fear of losing income or employment all together. For example, many participants mentioned that physical distancing was not actually possible, regardless of their employer's support for the measure. One participant compared her experience with airline procedures: “They tell you that when you stand in line to buy the flight, or to board the flight, to stay 1 meter away from each other, but inside the airplane they have 3 people crammed next to each other.”

Participants expressed that their employers told them how to protect themselves from COVID-19 infection but enforced policies that contradicted those procedures. One participant described how his employer would tell workers to take care of themselves while failing to implement basic precautions such as checking workers’ symptoms, encouraging sick employees to stay home, or suspending operations after a confirmed COVID-19 case among plant employees. Instead, this participant understood that the decision to take COVID-19 precautions “depends on each person: if you feel bad, well, better to not go [to work].”

Participants described that respecting the prescribed 6-foot physical distancing guidelines contradicted the production standards they felt were prioritized at their workplaces. One participant reasoned that “because [the employers] have to move their product, then they cannot remove a worker because the product would not turn out well, right? … they would have to modify the entire structure, the line, the company. Then it is not possible.”

Participants also felt obligated to work while sick, despite employers’ or other authorities’ recommendations to stay at home. One participant stated that “the employers care more about their production than their staff… if you are denied sick pay and you depend on your job, then you are not going to risk saying that I am sick.” Participants also expressed that they struggled to bring safety concerns to management for fear of retaliation. Others found that management was dismissive of workers’ complaints. Many participants also reported inadequate support from their supervisors and employers, that is, by not providing employees with healthcare benefits, pensions, training, safety measures, or communications channels or by lacking empathy. One participant said that she had lowered expectations of her employers’ response to COVID-19 because “they never do anything.” Another perceived that processing companies subordinated workers’ well-being in the interest of profit: “Plants do tell you, but it doesn't matter, they don't care. If you are sick, if you are healthy ... They don't care, what they care about is keeping their… profits.” In the words of another interviewee: “We are like a hamster… in a routine where they are exploiting us and trapping us. But they are profiting enormously.”

### Limited Agency

Many participants expressed a sense of limited agency in protecting themselves from COVID-19 or in controlling their life circumstances. At times, participants articulated this in an idiom of fate. For example, one participant said “When it is your time to die, it is your turn to die, it was your destiny. That disease is the same, if you have to get sick with coronavirus, then you have to get sick, whether you go outside or not.”

Other workers expressed that they felt economically compelled to keep working, despite facing known risks to the health and safety of themselves and their families. One worker explained that “It's just scary, scary from my point of view because I have a little baby. And she can easily get sick . . . But I have to make a check. I have to make decisions because I have bills to pay.” Many workers explained that they only continued to work at the processing plant through the COVID-19 pandemic because they had no other choice. One worker characterized his and other Mexican workers’ experiences in processing plants as being trapped in the industry as follows:

“We [Mexicans] do not leave because we are trapped in a cycle. They are not paying us what we're worth, they use us like puppets… Really, if you think about it, [if you leave] you're going to another job that is going to be the same… How are the laws there? Exactly like here. The bosses, the owners of the companies, are always going to be like everywhere else, squeezing you like an orange.”

### Perspectives of COVID-19 Vaccines

Because vaccine roll out started during data collection and the decision was made to include vaccine confidence questions after 9 interviews had taken place, only 31 of the 40 participants were asked about the COVID-19 vaccine. All participants who were asked about COVID-19 vaccines (*n*=31) expressed knowledge or awareness of 1 or more COVID-19 vaccines. However, only some participants were eager to take COVID-19 vaccines, whereas some others expressed an unwillingness to get vaccinated.

Some participants were uncertain, stating for example that they were unsure about or would “probably” take COVID-19 vaccines when available, although some of these participants said so believing that they would not have a choice. Participants uncertain about vaccination indicated that they would need more information before taking the vaccine, questioned the safety of the vaccine, or expressed that they would probably get it but had not yet decided. One interviewee said, “I was trying to see what the outcome would be first, know what I'm saying, first, so after a year later or something, I might want to get more information on that before I take it.” Another explained, “Because they say that there are side effects that affect you, the truth is, I don't feel motivated to [get the vaccine].”

Vaccine confidence varied by demographic group. For example, most Congolese participants who were asked about the COVID-19 vaccine expressed unwillingness to get vaccinated. The response of 1 participant suggests that vaccine hesitancy among Congolese persons is something that arose upon moving to the U.S. He explained, “I am from Africa, and I am used to vaccines. I used to take vaccines for malaria, polio but coming to America I hear people saying it's a way of government to control this, control that. I don't have a lot of information, but I know a lot of people are not open to it. We need to find out its side effects.”

Comparatively, no Central American participant asked about the COVID-19 vaccine was definitively unwilling to take the vaccine. Instead, they were split between those who were eager to take the vaccine and those who were uncertain; of larger concern was the availability of the vaccine. Most of the Central American participants were skeptical that the COVID-19 vaccine would even be made available to them. One interviewee insisted that Hispanic workers would figure out a way to obtain vaccines even if they were denied access, because “immigrants are smart and good at finding a way.”

Only 1 Mexican participant asked about the COVID-19 vaccine expressed that they were definitively unwilling to take the vaccine; most were eager, and some were uncertain. Most of the Black or African American workers expressed that they were unwilling to take the vaccine, citing concerns that it was too new to be trusted or the rumors that it caused COVID-19 or even death.

### Perceived Employer Efforts

Participants expressed receiving mixed messages about COVID-19 protections: they were told by their employers how to protect themselves but reported not being given the tools to do so or lacking the power to implement the recommendations. Many participants reported having the knowledge needed to prevent getting infected, but they reported not being able to navigate the congregate nature of job tasks and the perceived complacency of management in maintaining the availability of control measures in the workplace. Respondents perceived that employers and workplace policies represented barriers to COVID-19 protections at work. The data suggest that there is a need to strengthen enforcement of and oversight over engineering and administrative controls to keep workers safe, in addition to a need to ensure workers’ ability to consistently adopt all the recommended prevention measures. A recent study involving migrant and racial and ethnic minorities in nonstandard work arrangements suggests that encouragement from workplace management, supported by administrative policies such as paid sick leave,^15^ could potentially play a key role in mitigating COVID-19 transmission in the meat/poultry processing industry.

## DISCUSSION

The challenges workers reported in this study go beyond establishing a culture of safety. Participants feared losing their jobs if they stayed home sick, had limited access to sick leave or hazard pay, had no health or retirement benefits, and had no control over adjusting their work tasks to be consistent with COVID-19 safety recommendations. These barriers are consistent with those identified in previous research on workers belonging to racialized/ethnic minority groups, who are women, or who immigrated to the U.S. to find employment in jobs that can be categorized as nonstandard work arrangements.^22^ To reduce COVID-19 outbreaks in meat/poultry processing plants, it is critical to implement the hierarchy of controls through the use of engineering controls, administrative controls, and the use of personal protective equipment complemented by education and training.^9^

### Vaccine Confidence

The timing of the project afforded the opportunity to obtain perspectives on vaccine acceptance during the early onset of vaccine roll out. Despite awareness of COVID-19 vaccines among these essential workers, who became eligible to receive the vaccine early on, respondents expressed a wide range of interest and confidence in obtaining them. Central American participants were generally open to getting vaccinated but overwhelmingly anticipated that the vaccine would not be made available to immigrants, suggesting that they trusted the vaccine but did not trust that public health institutions or society at large would provide them with access to it. Conversely, some Congolese participants’ hesitancy toward immunization originated from exposure to misinformation after relocating to the U.S. These findings suggest that early vaccine eligibility for these essential workers was not enough. Efforts to promote vaccine confidence among these workers were also needed. These findings are supported by identified Centers for Disease Control and Prevention priority populations for improving health equity during the COVID-19 pandemic.^23^

All populations included in this project have faced historical and structural injustices leading to overrepresentation in underemployment and low-wage work as well as mistreatment ingrained in social, environmental, local, and governmental structures.^18^^,^^24^, ^25^, ^26^, ^27^, ^28^ The resulting mistrust in institutions, such as employers and government, could affect confidence in the then-new COVID-19 vaccines’ efficacy at preventing and mitigating severe acute respiratory syndrome coronavirus 2 (SARS-CoV-2) infection and its effects. Given participants’ active attempts to protect themselves where possible, COVID-19 vaccine hesitancy seemed to be more about mistrust of the vaccine than a potential lack of concern about the virus itself. Several studies found mistrust to be a key barrier to vaccine acceptance, and 1 study reported fewer vaccination sites in districts that had greater populations of Hispanic or Black communities.^18^ Conversely, a study of refugee communities found that a majority of respondents planned to get the vaccine, particularly if they were essential workers and male.^19^

Because participants reported that personally seeing or experiencing the effects of COVID-19 infection affected how seriously they took the virus, personal outreach could be vital to promoting vaccine confidence. A systematic review of international COVID-19 outbreaks in meat processing plants with migrant workers found that the most promising strategies for both workers and their communities are multiple approaches that include improved work site ventilation, infection prevention and control countermeasures, paid sick leave, and vaccination efforts that directly address workers’ concerns (in person, if possible) coupled with mandatory vaccination.^9^ We found that most of these approaches were reportedly lacking in the workplaces for the frontline workers we interviewed. Employers play an important role in implementing the hierarchy of controls for infection prevention and control through engineering controls (e.g., ensuring adequate ventilation), administrative controls (e.g., paid sick leave), and education and training of all workers (both supervisory and nonsupervisory) on infection prevention and control practices both employers and employees can adopt. The use of personal protective equipment (individual behavioral measures) is most effective when administered with the use of engineering and administrative controls as a foundation.

Additional efforts that could empower workers in the meat and poultry processing facilities and secure the necessary infection prevention and control measures could come from worker centers and labor organizations. Because meat and poultry processing facilities are located throughout the U.S. and different regions have varying levels of support or even existence of worker centers and labor organizations, improving the support meat and poultry processing employers provide may be best addressed at the community or regional level. Public health departments; labor agencies; and Occupational Safety and Health Administration regulators at the county, state, or regional level could routinely meet with the worker center and labor organizations to ensure that the labor and occupational safety and health needs of the meat and poultry processing workers were being met.

### Limitations

Due to limited sample size, the project did not focus on cross-population comparison. However, nuanced population-specific themes surrounding COVID-19 vaccine misinformation and skepticism emerged that pose potential implications for future work. Specifically, the responses of Congolese and Central American participants indicate that the migration experience can potentially affect vaccine confidence. Additionally, as this data was collected during the early days of the COVID-19 vaccine rollout it does not necessarily indicate the perspectives of meat/poultry processing workers at the time of this publication. However, the data does elucidate how structural factors, migration history, and socially trusted sources of information may affect initial vaccine rollout and uptake during public health emergencies.

## CONCLUSIONS

Linguistic, racial, and ethnic minorities are often considered hard to reach and are underrepresented in the public health literature.^29^ The inclusion of Congolese refugees and indigenous Central American and Mexican migrants in this study not only helps to address these gaps but also highlights the feasibility of including a greater breadth of linguistic minorities in public health research. Public health organizations can develop the capacity to work with linguistic minorities by building relationships with community-based organizations. This aligns with previous literature clarifying that these populations can indeed be reached if the appropriate institutional capacity is developed.^29^^,^^30^ A central lesson of the COVID-19 pandemic is the need to develop a more inclusive public health system that all communities are shown to belong to. Converting collaborations with community-based organizations; worker centers; labor organizations; health departments; and Occupational Safety and Health Administration offices that arose during the pandemic into long-term, sustainable relationships is an essential step to developing the institutional capacity of public health institutions to address long-standing health inequities.

Meat/poultry processing facilities have played a key role in disease transmission during the COVID-19 pandemic.^1^^,^^2^^,^^31^ This has presented an opportunity to emphasize the importance of occupational safety and health, including the role of a hierarchy of controls in infection prevention and control alongside workplace vaccination programs, within an industry already known to need greater protections at work.^6^, ^9^, ^32^ The study results contribute to the health equity literature by highlighting the relationship between migration, work, and health inequities and the potential role of work in facilitating or hindering public health efforts among these essential workers and their diverse communities.^33^^,^^34^ It is vital that both a health equity and occupational safety and health perspective be prioritized in research exploring the intersection of the workplace, communities, and public health to optimize health and safety promotion efforts in the workplace.

## CRediT authorship contribution statement

**Jacqueline M. Sivén:** Conceptualization, Methodology, Formal analysis, Resources, Writing – original draft, Writing – review & editing, Supervision, Funding acquisition. **Julia F. Coburn:** Formal analysis, Investigation, Data curation, Writing – original draft, Writing – review & editing, Project administration. **Tristan P. Call:** Formal analysis, Investigation, Resources, Writing – original draft, Writing – review & editing. **Dillon Mahoney:** Formal analysis, Investigation, Resources, Writing – original draft, Writing – review & editing. **Rebeca Rodríguez Flores:** Formal analysis, Investigation, Resources. **Harpriya Kaur:** Formal analysis, Writing – review & editing. **Michael A. Flynn:** Conceptualization, Writing – review & editing. **Cammie K. Chaumont Menéndez:** Conceptualization, Formal analysis, Writing – original draft, Writing – review & editing.
